# ALKBH1 Is a Histone H2A Dioxygenase Involved in Neural Differentiation

**DOI:** 10.1002/stem.1228

**Published:** 2012-09-07

**Authors:** Rune Ougland, David Lando, Ida Jonson, John A Dahl, Marivi Nabong Moen, Line M Nordstrand, Torbjørn Rognes, Jeannie T Lee, Arne Klungland, Tony Kouzarides, Elisabeth Larsen

**Affiliations:** a Centre for Molecular Biology and Neuroscience, Institute of Medical Microbiology, Oslo University Hospital, Rikshospitalet and University of OsloOslo, Norway; b Gurdon Institute and Department of Pathology, University of CambridgeCambridge, United Kingdom; c Department of Molecular Biology, Massachusetts General Hospital, Howard Hughes Medical InstituteBoston, Massachusetts, USA; d Department of Genetics, Harvard Medical SchoolBoston, Massachusetts, USA

**Keywords:** ALKBH1, Stem cells, Histone dioxygenase, Neural development, Epigenetics

## Abstract

AlkB homolog 1 (ALKBH1) is one of nine members of the family of mammalian AlkB homologs. Most *Alkbh1^−/−^* mice die during embryonic development, and survivors are characterized by defects in tissues originating from the ectodermal lineage. In this study, we show that deletion of *Alkbh1* prolonged the expression of pluripotency markers in embryonic stem cells and delayed the induction of genes involved in early differentiation. In vitro differentiation to neural progenitor cells (NPCs) displayed an increased rate of apoptosis in the *Alkbh1^−/−^* NPCs when compared with wild-type cells. Whole-genome expression analysis and chromatin immunoprecipitation revealed that ALKBH1 regulates both directly and indirectly, a subset of genes required for neural development. Furthermore, our in vitro enzyme activity assays demonstrate that ALKBH1 is a histone dioxygenase that acts specifically on histone H2A. Mass spectrometric analysis demonstrated that histone H2A from *Alkbh1^−/−^* mice are improperly methylated. Our results suggest that ALKBH1 is involved in neural development by modifying the methylation status of histone H2A. Stem Cells 2012;30:2672–2682

## INTRODUCTION

Embryonic stem cells (ESCs) possess unique abilities to self-renew indefinitely and differentiate into all of the cell types of the three germ layers [1, 2]. The regulatory networks that control ESC pluripotency and self-renewal are key features in development.

The ability of ESCs to self-renew and differentiate requires a considerable degree of epigenetic plasticity. Genes crucial for pluripotency are rapidly silenced by histone modifications and DNA methylation during early differentiation, whereas genes that are required later in cellular differentiation are held in a transient repressed state by bivalent chromatin marks that are easily removed to activate transcription [3]. The maintenance of pluripotency is largely governed by the transcription factors OCT4, NANOG, and SOX2 [4–6]. Genome-wide studies have shown that these core factors occupy the promoters of actively transcribed genes encoding transcription factors and chromatin-modifying enzymes that promote ESC self-renewal. They also occupy the promoters of genes encoding regulators that are silent in ESCs but expressed during lineage commitment and subsequent differentiation [7, 8]. Although changes in DNA methylation and histone modification have been intensively explored during the earliest stages of differentiation of pluripotent cells, the mechanisms by which histone modifications influence pluripotency and development remain unknown [9].

Some years ago, the AlkB homolog 1 (*ALKBH1*) promoter was shown to be bound by NANOG and OCT4 in human ESCs (hESCs) [7], which implies that these key factors may regulate *ALKBH1* expression. ALKBH1 is one of nine members of the newly discovered family of AlkB hydroxylases in mammals. The AlkB protein from *Escherichia coli* (*E. coli*) is a DNA repair enzyme that uses Fe(II) and 2-oxoglutarate (2OG) to hydroxylate the methyl groups associated with certain forms of DNA damage [10, 11]. The hydroxymethyl group is unstable and is spontaneously released as formaldehyde, resulting in the removal of the methyl group from DNA. The high degree of conservation of the AlkB sequence throughout evolution and across kingdoms suggests that AlkB homologs play an important biological role [12]. Of the nine mammalian homologs of AlkB, only four have been assigned enzymatic functions. Whereas ALKBH2 and ALKBH3 are DNA or RNA repair enzymes [13–15], ALKBH8 is involved in epigenetic regulation through its ability to modify tRNA [16, 17] and ALKBH9 (also called alpha-ketoglutarate-dependent dioxygenase (FTO)) demethylates 6-methyladenine in nuclear RNA to adenine [18]. The in vivo functions of the remaining ALKBH proteins remain elusive. In vitro, recombinant ALKBH1 can both demethylate 3-methylcytosine [19] and display DNA lyase activity [20]. It remains unclear, however, whether these two activities are physiologically relevant, and ALKBH1 is generally considered to be a nuclear enzyme with a role in epigenetic regulation [21–23]. Histone demethylases with JmjC domains use the same mechanism as AlkB to remove methyl groups from histones (Fig. 6A) [24]. Based on this shared mechanism and a protein binding motif in ALKBH1 potentially involved in binding of histones [25], it has been speculated that ALKBH1 might be a histone demethylase [11]. Recently, we showed that *Alkbh1* mutant mice display a complex phenotype with abnormalities in tissues originating from the ectodermal lineage, including neural tube defects such as exencephaly and spina bifida [26]. Approximately 10% of *Alkbh1^−/−^* mice appear relatively normal, whereas the most affected mice die during early embryogenesis. These findings indicate a key role for ALKBH1 in early development.

Here, we report that homozygous disruption of *Alkbh1* in mouse ESCs leads to the sustained expression of pluripotency markers upon differentiation and the delayed induction of neuroectodermal genes. In vitro differentiation to neural progenitor cells (NPCs) displayed an increased rate of apoptosis in the *Alkbh1^−/−^* NPCs when compared with wild-type (WT) cells. We identify genes bound and regulated by ALKBH1 using chromatin immunoprecipitation followed by high-throughput sequencing (ChIP-seq). The majority of these genes are involved in early neural development. Furthermore, in vitro enzyme activity assays and mass spectrometric analysis of histones from *Alkbh1^−/−^* cells indicate that ALKBH1 is a histone dioxygenase that acts specifically on histone H2A. Our results suggest that ALKBH1 is involved in the epigenetic regulation of neural development by modifying the methylation status of histone H2A.

## MATERIALS AND METHODS

### Derivation, Culture, and Differentiation of Mouse ESC

Mouse ESCs were established as described previously [51] and cultured in Knockout Dulbecco's modified Eagle's media (KO-DMEM) (Invitrogen 10829-018, Carlsbad, CA, USA, http://www.lifetechnologies.com) supplemented with 20% KO serum replacement (Invitrogen 10828-028), 100 U/ml Penicillin-Streptomycin (Invitrogen 15140-122), 0.1 mM nonessential amino acids (Invitrogen 11140-035), 2 mM GlutaMAX (Invitrogen 35050-038), 0.1 mM 2-mercaptoethanol (Sigma M7522, St. Louis, MO, USA, http://www.sigmaaldrich.com), and 1,000 U/ml leukemia inhibitory factor (LIF) (Millipore ESG1107, Billerica, MA, USA, http://millipore.com). All ESC cultures were grown on a layer of irradiated CF-1 MEFs (Globalstem GSC-6001G, Rockville, MD, USA, http://www.globalstem.com) on gelatin-coated plates. Differentiation was induced by adding 1 μM of all-*trans* retinoic acid (Stemgent 04-0021, Cambridge, MA, USA, https://www.stemgent.com) and removing LIF. Proliferation and viability were assessed using the Countess Automated Cell Counter (Invitrogen C10227) with trypan blue. Neural differentiation was induced sequentially; EBs were formed by aggregation in suspension culture for 4 days followed by treatment with all-*trans* retinoic acid for 4 days. EBs were then plated onto gelatin-coated plates and propagated in ITSFn medium (DMEM/F12 [Invitrogen 31330-095] containing 1× Insulin-Transferrin-Selenium-G Supplement [Invitrogen 41400-045], and fibronectin at 2.5 μg/ml [Invitrogen 33010-018]). After 5 days, the cells were dissociated into single cells and replated onto polyornithine-coated plates in B27/N2 medium (Neurobasal-A Medium [Invitrogen 10888-022]) containing 1× B27 (Invitrogen 17504-044), 0.5× N2 (Invitrogen 17502-048), 1 μg/ml laminin (Sigma L2020), and 10 ng/ml fibroblast growth factor 2 (FGF2) (Milteny Biotec 130-093-841, Bergisch Gladbach, Germany, http://www.miltenyibiotec.com). Cells were further induced toward the neural lineage by withdrawal of FGF2.

### CO_2_ Capture Assay

Hydroxylation activity was determined radiochemically by measuring hydroxylation-dependent release of [^14^C]CO_2_, as described previously [52]. Standard assay conditions comprised 40 μl reactions containing 30 mM Hepes (pH = 7.5), 90 μM 2OG, 10 μM [1-^14^C]2OG (PerkinElmer Life Sciences, Waltham, MA, USA, http://www.perkinelmer.com), 4 mM ascorbate, 250 μM (NH_4_)_2_Fe(SO_4_)_2_, purified ALKBH1 or ALKBH1 H228A, and substrate. For each set of assays, two stocks were made. The first, with a total volume of 20 μl, contained substrate. The second contained purified enzyme and all other reagents. Assays were started by the addition of 20 μl freshly prepared enzyme stock to the substrate stock. To recover [^14^C]CO_2_, a strip of Whatman 3MM filter paper, which had been presoaked in 30 mM calcium hydroxide, was immediately inserted into the neck of the tube, and the tube was sealed. The assays were then incubated at 37°C for 60 minutes. Upon reaction completion, filter strips were removed, air-dried, treated with scintillant, and then counted for radioactivity in a scintillation counter. We then incubated 1 μg of purified ALKBH1 or ALKBH1 H228A with 10 μg of either purified HeLa core histones or core histones immunodepleted of H2A or H3. The ALKBH1 H228A variant is an inactive protein harboring a mutation in the iron-binding domain. Mutation of histidine 228 in ALKBH1 to alanine was carried out using the QuickChange site-directed mutagenesis kit (Stratagene 200518, Santa Clara, CA, USA, http://www.genomics.agilent.com). To prepare histone H2A and H3 immunodepleted samples, we incubated 100 μg of HeLa core histones overnight with 25 μg of H2A antibody (Abcam ab13923, Cambridge, United Kingdom, http://www. abcam.com) or H3 antibody (Abcam ab1791). The next day, antibody-bound histone was removed using protein-A Sepharose.

### Immunofluorescent Staining of ESCs

Cultures of hESCs were trypsinized, and the single-cell suspension was cytospun onto Superfrost Plus slides (Thermo Scientific 4951PLUS, Waltham, MA, http://www.thermofisher.com). Mouse ESCs were cultured on coverslips dispersed in 10 cm Petri dishes or cytospun onto Superfrost Plus slides. For hESCs, the following antibodies were used: rabbit anti-ALKBH1 (Abcam ab18525; 1:500), mouse anti-OCT4 (BD Biosciences 611202; 1:200, San Jose, CA, USA, http://www.bdbiosciences.com), and mouse antimitochondria [MTC02] (Abcam ab3298; 1:500). For mouse ESCs, the following antibodies were used: rabbit anti-NUCLEOPHOSMIN (Abcam ab15440; 1:100–1:1,000), rabbit anti-PML (Progressive Multifocal Leukoencephalopathy) (Millipore AB1370; 1:200–1:500), and mouse anti-NESTIN (Millipore MAB353; 1:200). Secondary antibodies used are Invitrogen Alexa Fluor 488 goat anti-rabbit A-11034 and/or Invitrogen Alexa Fluor 555 goat anti-mouse A-21424. All microscopy were done using an Axio Observer.Z1 microscope (Carl Zeiss, Thornwood, NY, USA, http://www.zeiss.com).

### Apoptosis Assays

The TUNEL assay (Roche Applied Science 12156792910, Indianapolis, IN, https://www.roche-applied-science.com) was performed according to manufacturer's instructions. For flow cytometric analysis, mESCs were stained with the Dead Cell Apoptosis Kit with Annexin V Alexa Fluor 488 and propidium iodide (PI) (Invitrogen V13241) according to manufacturer's instructions. Analyses were performed on a BD Accuri C6 Flow Cytometer using the BD Accuri CFlow software. A minimum of 10,000 cells were analyzed per data point.

### RT-PCR Analysis and TaqMan Low-Density Arrays

Total RNA was purified using the miRNeasy mini kit (Qiagen 217004, Valencia, CA, USA, http://www.qiagen.com) according to the manufacturer's instructions. Any DNA remnants were removed using amplification-grade DNase 1 (Invitrogen 18068-015), and cDNA was made using the High Capacity cDNA Reverse Transcription Kit (Applied Biosystems 4368814, Carlsbad, CA, USA, http://www.appliedbiosystems.com). TaqMan Mouse Stem Cell Pluripotency Array v.2 (Applied Biosystems 4385363) analysis was performed using an Applied Biosystems 7900HT Fast Real-Time PCR System. Genes with a Ct value >32 were removed and considered not expressed. The RT-PCR reactions were carried out using a StepOnePlus instrument. For overexpression of ALKBH1 in *Alkbh1^−/−^* cells, mouse *Alkbh1* was cloned into pCl-Neo (Promega E1841, Fitchburg, WI, http://www.promega.com/) and the construct was transfected into mESCs using FuGene (Roche 815 091 001) at a FuGene/DNA ratio of 6:1 (μl/μg). The transfected cells were cultured for 8 days in media supplemented with 500 μg/ml of G-418 (Invitrogen 10131) prior to RNA purification and real-time PCR analysis.

### ChIP-Seq and Microarray Analysis

Total RNA was extracted from mESCs using a miRNeasy mini kit (Qiagen 217004) according to the manufacturer's protocol. Quality was verified using an Agilent Bioanalyzer 2100 instrument (RIN value (RNA integrity number) between 9.5 and 10.0). The whole-genome gene expression profiling was performed using an Affymetrix GeneChip Mouse Genome 430 2.0 Array (Cleveland, OH, http://www.affymetrix.com). Affymetrix raw data were generated using GCOS 1.4 (GeneChip Operating Software, Affymetrix), and the signal intensities of each probe set were normalized using the robust microarray analysis algorithm. We used a *t* test with randomized variance (5% FDR correction) to identify differentially expressed genes. Class comparison analysis was used to identify genes for which (a) the signal in one group was always (i.e., for each the triplicate assays) higher or lower than for the other group, and (b) the fold difference in expression levels was ≥1.5-fold (log_2_). GO classification was done using TopGO (Bioconductor). The ChIP experiments were performed on hESCs (H9) using a mouse monoclonal anti-ALKBH1 (Sigma A8103). According to standard ChIP procedures, cells were exposed to formaldehyde to covalently cross-link protein to DNA and protein to protein to conserve interactions that are within 2 Å. Sequencing was done using an Illumina/Solexa Genome Analyzer IIx platform. Analysis was done as described previously [53]. All microarray and ChIP-seq data from this study have been submitted to the Gene Expression Omnibus (GEO) database (GSE30561).

### Mass Spectrometry

Histones were purified and separated as described in Shechter et al. [54]. The H2A band was excised, digested with chymotrypsin and/or Asp-N, and analyzed by mass spectrometry using an LTQ-Orbitrap from Thermo Electron (Taplin MS Facility, Harvard University, Boston, MA [https://taplin.med.harvard.edu/home]).

## RESULTS

### ALKBH1 Is Located in the Nucleus

**Figure 1:**
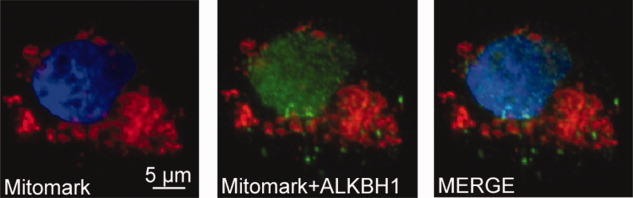
The ALKBH1 protein localizes to the nuclei of human embryonic stem cells (ESCs). Staining of human ESCs (H9) revealed that ALKBH1 is predominantly nuclear. The mitomarker is red (Alexa-555), ALKBH1 is green (Alexa-488), and 4′,6–diamidino–2–phenylindole (DAPI) is blue. Abbreviation: ALKBH1, AlkB homolog 1.

The cellular localization of ALKBH1 has been a matter of debate [19, 22]. Whereas Pan and colleagues reported in 2007 that mouse ALKBH1 is a nuclear protein that localizes to euchromatin, a year later, Westby and colleagues found human ALKBH1 in mitochondria and suggested that ALKBH1 was involved in the repair of mitochondrial DNA. We used a human cell line (H9) cultured under conditions favoring pluripotency and self-renewal, and performed a double immunofluorescence analysis using an anti-ALKBH1 antibody and an antibody that recognizes a 60-kDa nonglycosylated protein component of mitochondria found in human cells. In the hESC line, ALKBH1 localized predominantly to the nucleus, which is consistent with its proposed role in epigenetic regulation (Fig. 1). Only a very small fraction of ALKBH1 was found outside the nucleus, and these scarce sites of ALKBH1 accumulation did not colocalize with mitochondria.

### Alkbh1 Is Dispensable for mouse ESC Self-Renewal, but Alkbh1-Deficiency Delays Differentiation

**Figure 2:**
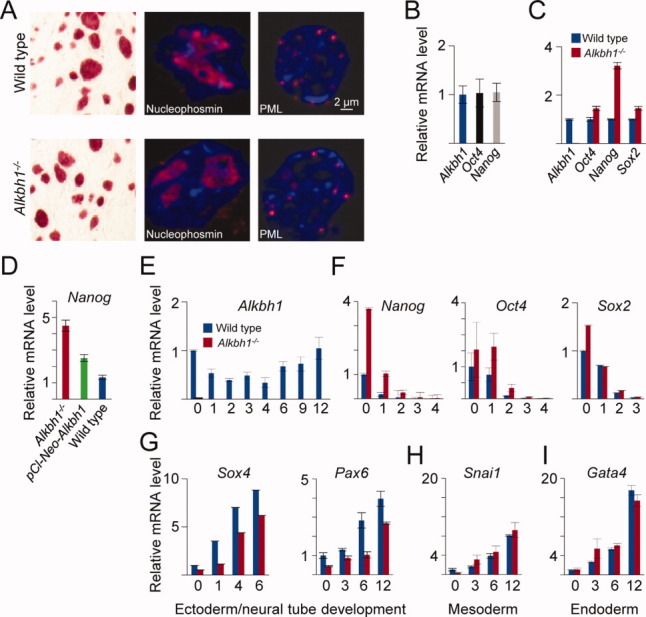
Differentiation is delayed in *Alkbh1^−/−^* embryonic stem cells (ESCs). **(A):** Alkaline phosphatase (AP) staining of wild-type (WT) and *Alkbh1^−/−^* mouse ESCs (mESCs). *Alkbh1^−/−^* mESCs express markers of pluripotency. **(B):** The measurement of *Alkbh1* expression (blue bar) in undifferentiated mESCs showed expression at the same level as the core pluripotency transcription factors (represented by *Oct4* and *Nanog*). **(C):** Quantitation of the master regulators of pluripotency revealed upregulation of genes encoding all three transcription factors (*Oct4, Nanog*, and *Sox2*) in undifferentiated *Alkbh1^−/−^* mESCs. The *Nanog* gene displayed the greatest (more than fourfold) increase in expression. **(D):** Quantitative real-time PCR (qRT-PCR) analysis showed that expression of ALKBH1 in *Alkbh1^−/−^* mESCs (pCl-*Neo*-*Alkbh1*) reduced *Nanog* expression, albeit not to levels as low as those found in WT mESCs. **(E):** The qRT-PCR analysis of WT mESCs that were forced to differentiate along an ectodermal path (i.e., by incubation in media without leukemia inhibitory factor and supplemented with 1 μM all-*trans* retinoic acid) revealed that *Alkbh1* was downregulated during the first 4 days of differentiation, before it was upregulated. **(F):** As expected, levels of expression of the pluripotency markers *Oct4*, *Sox2*, and *Nanog* decreased, albeit at a slower rate in *Alkbh1^−/−^* mESCs than in WT mESCs, suggesting delayed differentiation of *Alkbh1^−/−^* mESCs. **(G):** The impeded differentiation of *Alkbh1^−/−^* mESCs was confirmed by demonstrating the induction of *Sox4* and *Pax6* at a later time in the *Alkbh1^−/−^* mESCs than in WT mESCs. **(H):** Expression of the mesodermal marker *Snai1* was identical in *Alkbh1^−/−^* mESCs and WT mESCs. **(I):** Expression of the endodermal marker *Gata4* was identical in *Alkbh1^−/−^* mESCs and WT mESCs. Vertical lines represent the 1 ± SEM. Abbreviations: ALKBH1, AlkB homolog 1; PML, promyelocytic leukemia protein.

Approximately 80% of *Alkbh1^−/−^* embryos died during early development and was resorbed shortly after conception. We have previously shown that ALKBH1 is predominantly expressed during embryonic development and in adult testis, brain, and eye [26]. To further dissect the role of ALKBH1 upon differentiation, we derived *Alkbh1^−/−^* mouse ESCs (ESCs) from mouse embryos 3.5 days after gestation (E3.5) (supporting information Fig. S1). The WT and *Alkbh1^−/−^* mESCs in this study are derived from littermate E3.5 embryos after mating of heterozygous mice. The ability to either propagate ESCs in vitro in a pluripotent and self-renewing state or induce them to differentiate into any of the three germ layers [27, 28] makes them suitable for investigating lineage commitment and early differentiation. Over the course of >15 passages, mutant cells maintained a normal undifferentiated ESC morphology and stained positive for alkaline phosphatase (Fig. 2A). Data from quantitative real-time PCR (qRT-PCR) showed that the *Alkbh1* mRNA level in WT mESCs grown under self-renewing conditions equaled the expression of representative pluripotency markers (*Oct4* and *Nanog*) (Fig. 2B). Intriguingly, the transcript levels of *Oct4*, *Nanog*, and *Sox2* were higher in the *Alkbh1^−/−^* mESCs than in WT mESCs (Fig. 2C). Level of *Nanog* transcripts was as much as fourfold higher in *Alkbh1^−/−^* mESCs than in WT mESCs, suggesting that *Alkbh1^−/−^* mESCs are refractory to differentiation. To test whether *Alkbh1* expression is required for normal levels of *Nanog* expression, we expressed *Alkbh1* in *Alkbh1^−/−^* mESCs. The mouse *Alkbh1* gene was cloned in a pCl-*Neo* vector, transfected into *Alkbh1^−/−^* mESCs, and cultured for 8 days in the presence of G-418. As shown in Figure 2D, qRT-PCR analysis revealed that *Nanog* expression was much lower in the pCl-*Neo*-*Alkbh1* cells than in *Alkbh1^−/−^* mESCs, albeit still higher than in WT mESCs. This indicates an involvement of ALKBH1 in regulating NANOG levels in mESCs.

Most *Alkbh1^−/−^* mice that survived to the time of birth are characterized by aberrant differentiation along the neuroectodermal lineage. Thus; we forced mESCs to differentiate toward the ectodermal lineage by induction with retinoic acid for 12 days. In WT mESCs, *Alkbh1* mRNA levels decreased initially upon induction with retinoic acid (until day 4), and then increased until day 12, suggesting a role for ALKBH1 in differentiation and development (Fig. 2E). Although expression of *Sox2*, *Oct4*, and *Nanog* were silenced in both cell lines by day 3, *Alkbh1^−/−^* mESCs had higher levels of *Sox2*, *Nanog*, and *Oct4* transcripts during the first 2 days after induction with retinoic acid, which are indicative of impeded differentiation (Fig. 2F). This effect of *Alkbh1* deficiency was further confirmed by demonstrating that lineage-specific genes from all three germ layers were expressed in WT mESCs after 1 day of differentiation, whereas the neuroectodermal lineage was induced less extensively in *Alkbh1^−/−^* cells. Specifically, the induction of the SOX4 transcription factor and paired box protein 6 (PAX6) (Fig. 2G) was impaired in *Alkbh1^−/−^* mESCs. The SOX4 protein plays a central role during neuronal maturation and in ensuring the survival of neuronal cells [29–31]. The PAX6 protein is crucial for the development of eyes and tissues derived from the ectodermal lineage [32]. Representative transcript markers of mesodermal and endodermal tissues were not affected by *Alkbh1* targeting (Fig. 2H, 2I). These data suggest that ALKBH1 is involved in the early differentiation along the neuroectodermal lineage. This notion is underscored by the phenotype of *Alkbh1^−/−^* mice.

### Loss of *Alkbh1* Leads to Increased Apoptosis

**Figure 3:**
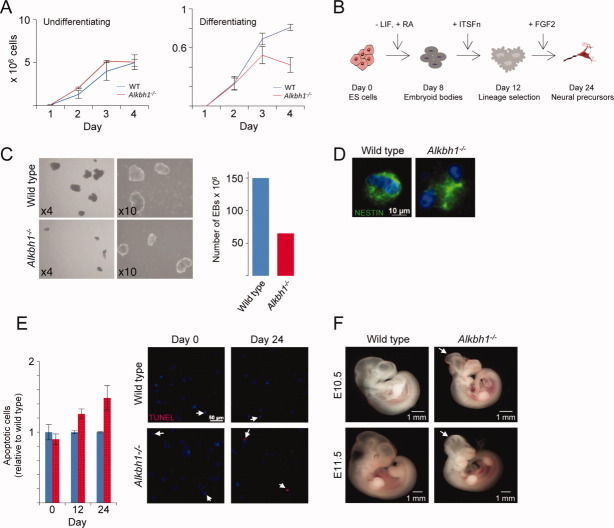
Differentiation of *Alkbh1^−/−^* mouse ESCs leads to increased apoptosis. **(A):** Proliferation rate of mouse ESCs cultured under conditions favoring self-renewal showed no difference between WT and *Alkbh1^−/−^*. Upon induction of differentiation, the culture of *Alkbh1^−/−^* mouse ESCs had a slower growth rate and increased cell death from differentiation day 3. **(B):** Timeline showing the steps of the neural differentiation protocol of mouse ESCs. **(C):***Alkbh1^−/−^* mouse ESCs form embryoid bodies that are fewer and smaller than their WT counterpart. **(D):** After 24 days of differentiation, the mouse ESCs formed NESTIN-positive neural progenitor cells (NPCs). **(E):** TUNEL staining reveals an increased rate of apoptosis in the NPCs derived from *Alkbh1^−/−^* mESCs. **(F):** Ten percentage of E10.5-E11.5 *Alkbh^−/−^* embryos exhibits neural tube defects (white arrowheads) when compared with WT littermates [26]. Abbreviations: ALKBH1, AlkB homolog 1; ESC, embryonic stem cell; FGF2, fibroblast growth factor 2; LIF, leukemia inhibitory factor; RA, retinolic acid; WT, wild type.

To further investigate the differentiation defect in the *Alkbh1^−/−^* ESCs, we characterized different aspects of differentiation (proliferation, cell death, and differentiation phenotype). Proliferation rate was analyzed by counting cells over several days (Fig. 3A). Under self-renewal conditions, there was no difference in proliferation rate and viability between the WT and the *Alkbh1^−/−^* ESCs. However, when cultured in a feeder-free manner in the presence of retinoic acid, the *Alkbh1^−/−^* culture was clearly less proliferative during the first days of differentiation. This reduction is to a certain extent explained by a decline in viability (supporting information Fig. S2). We further differentiated WT and *Alkbh1^−/−^* ESCs toward NPCs and analyzed the cells at different time points (8, 12, and 24 days) (Fig. 3B). After 8 days, the WT and *Alkbh1^−/−^* cells had formed embryoid bodies (EBs). The number of *Alkbh1^−/−^* EBs was 50% compared to WT EBs and they were considerably smaller (Fig. 3C). After 24 days, WT and *Alkbh1^−/−^* NPCs expressed the neural marker NESTIN (Fig. 3D). TUNEL staining of *Alkbh1^−/−^* NPCs showed increased apoptosis compared to WT (Fig. 3E). Quantification of apoptosis was done by fluorescence-activated cell sorting analysis after staining for ANNEXIN V. After 12 days of differentiation, the *Alkbh1^−/−^* NPCs showed a 30% increase of apoptotic cells relative to the WT NPCs. This increase in apoptosis raised to approximately 50% after 24 days of differentiation (Fig. 3E). Taken together, our results indicate that *Alkbh1* deficiency leads to increased apoptosis during neural development. Programmed cell death occurs during normal CNS development [33], and the neural tube defects observed in the *Alkbh1^−/−^* embryos could be due to increased apoptotic cell death (Fig. 3F).

### ALKBH1 Regulates Neural Development

**Figure 4:**
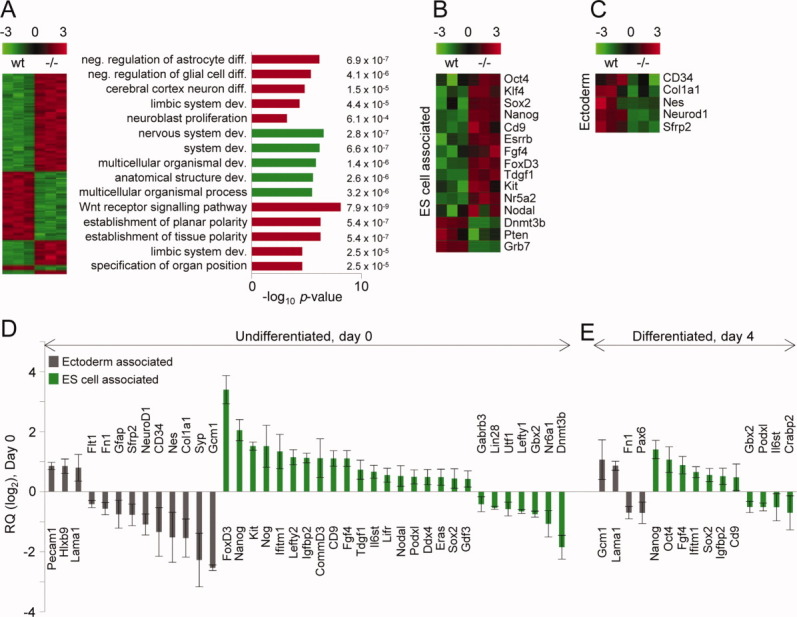
*Alkbh1*^−/−^ mouse ESCs show severe dysregulation of genes involved in differentiation and pluripotency. **(A):** An Affymetrix GeneChip Mouse Genome 430 2.0 Array revealed that 248 transcripts were differentially expressed between WT and *Alkbh1^−/−^* mouse ESCs (fold change >1.5 [log_2_], false discovery rate (FDR) 5%). Five discrete clusters were identified by visual inspection. Gene ontology classification according to biological process (TopGO, Bioconductor) showed an enrichment of terms related to differentiation, the development of the nervous system, and Wnt signaling. **(B):** Selected ESC-related genes and genes related to pluripotency. **(C):** Selected genes related to ectodermal development. **(D):** Analysis involving the TaqMan Mouse Stem Cell Pluripotency Array v.2 (Applied Biosystems) was performed to compare the expression of lineage marker genes and pluripotency-associated genes in WT and *Alkbh1^−/−^* mouse ESCs grown under self-renewal conditions. The *Gapdh* gene was used as an internal control. Selected genes with Ct <32 were included. Gray, ectoderm-associated genes; green, pluripotency- and ESC-associated genes. **(E):** As in (D), but with mESCs allowed to differentiate for 4 days. Vertical lines represent the 1 ± SEM. Abbreviations: ESC, embryonic stem cell; WT, wild type.

To better understand the dysregulation caused by *Alkbh1* deficiency, we performed genome-wide expression microarray analysis on undifferentiated mESCs. Whereas 157 genes were upregulated in *Alkbh1^−/−^* mESCs relative to WT mESCs, 91 genes were downregulated (Fig. 4A). These calls were based on a >1.5-fold difference in abundance and *p* < .05. Hierarchical clustering of the differentially expressed genes revealed five discrete clusters of transcripts commonly regulated during the differentiation of mESCs. Two of these clusters contain genes expressed at a higher level in *Alkbh1^−/−^* mESCs than in WT mESCs (upregulated genes), whereas three encode transcripts less abundant in *Alkbh1^−/−^* mESCs than in WT mESCs (downregulated genes). Gene ontology (GO) analysis indicated that upregulated genes in cluster one are involved in differentiation along the neural lineage, in addition to several key markers of pluripotency. Again we identified upregulation of several key markers of undifferentiated mESCs, including *Nanog*, *Oct4*, and *Sox2* (Fig. 4B). The majority of downregulated genes in cluster two are involved in the development and differentiation of the ectodermal lineage (Fig. 4C), whereas upregulated genes in cluster three are involved in the establishment of polarity and Wnt receptor signaling. The remaining two clusters contain too few transcripts for GO analysis. We confirmed the data generated by the microarray analysis using a TaqMan Mouse Stem Cell Pluripotency Array v.2, containing probes specific for 96 transcripts (Fig. 4D). In accordance with previous results, genes involved in pluripotency and self-renewal were upregulated in the *Alkbh1^−/−^* mESCs relative to WT mESCs. Additionally, compared with WT mESCs, gene expression in the ectodermal lineage was repressed in *Alkbh1^−/−^* mESCs, whereas genes implicated in differentiation along mesodermal, endodermal, and trophectodermal lineages were unaffected. Consistent with previous results, we found elevated expression of ESC-specific genes, including *Oct4*, *Nanog*, and *Sox2*, in *Alkbh1^−/−^* mESCs differentiated for 4 days (Fig. 4E).

### Identification of ALKBH1 Target Genes

**Figure 5:**
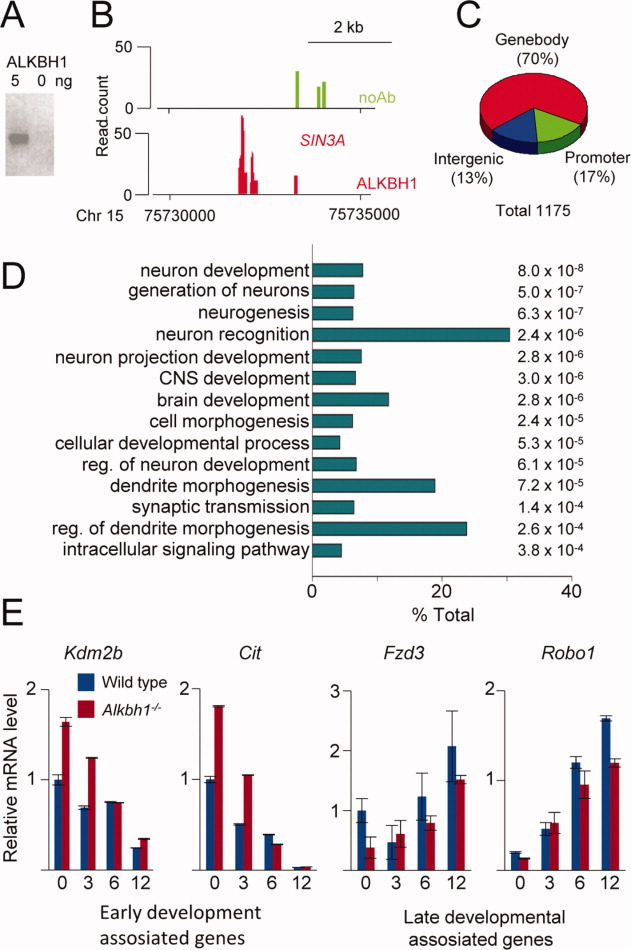
ChIP-seq analysis reveals that ALKBH1 occupies genes that encode developmental regulators in human embryonic stem cells. **(A):** The specificity of the antibody used in the ChIP-seq assay was confirmed by Western blotting of recombinant human ALKBH1 protein. **(B):** Image from the UCSC Genome Browser (http://genome.ucsc.edu/). This view of the paired amphipathic helix protein (SIN3A) illustrates the read distribution over one ALKBH1-binding region designated as a read. The locations of the amplicons detected by ChIP-qPCR are indicated at the bottom. Green bars represent reads from the control sample lacking antibody, whereas red bars represent reads from the ALKBH1 immunopreciptated sample. **(C):** Analysis of the distribution of ALKBH1 reads over various genomic features revealed that the majority of reads reside in the gene body (70%), whereas 13% and 17% are associated with intergenic and promoter regions, respectively. **(D):** Gene ontology analysis of genes occupied by ALKBH1 revealed a significant enrichment for regulators of neural development. **(E):** Analysis by quantitative real-time PCR of a selection of the occupied genes confirmed that ALKBH1 regulates their expression. Vertical lines represent the 1 ± SEM. Abbreviation: ALKBH1, AlkB homolog 1.

To identify genes that are bound by ALKBH1, we performed ChIP-seq using a human ALKBH1-specific antibody (Fig. 5A). In our hands, the mouse ALKBH1-specific antibody did not work. The ChIP-seq experiments revealed 1,175 ALKBH1-occupied genomic regions in hESCs. Representative ChIP-seq data, shown for sequencing reads within *SIN3A*, are shown in Figure 5B. The ChIP-seq results were confirmed by ChIP-qPCR (supporting information Fig. S3). Notably, no ALKBH1 sequencing reads were localized to mitochondrial DNA. This supports the observed nuclear localization of ALKBH1 (Fig. 1). Roughly 70% of all sequencing reads was located in the gene body; 13% was intergenic, and 17% was in promoter regions (Fig. 5C). Therefore, 87% of the sequencing reads correlated with protein coding genes. This distribution suggests a gene-related function for ALKBH1 and implicates a possible regulatory role for ALKBH1 that involves the targeting of specific genes.

**Figure 6:**
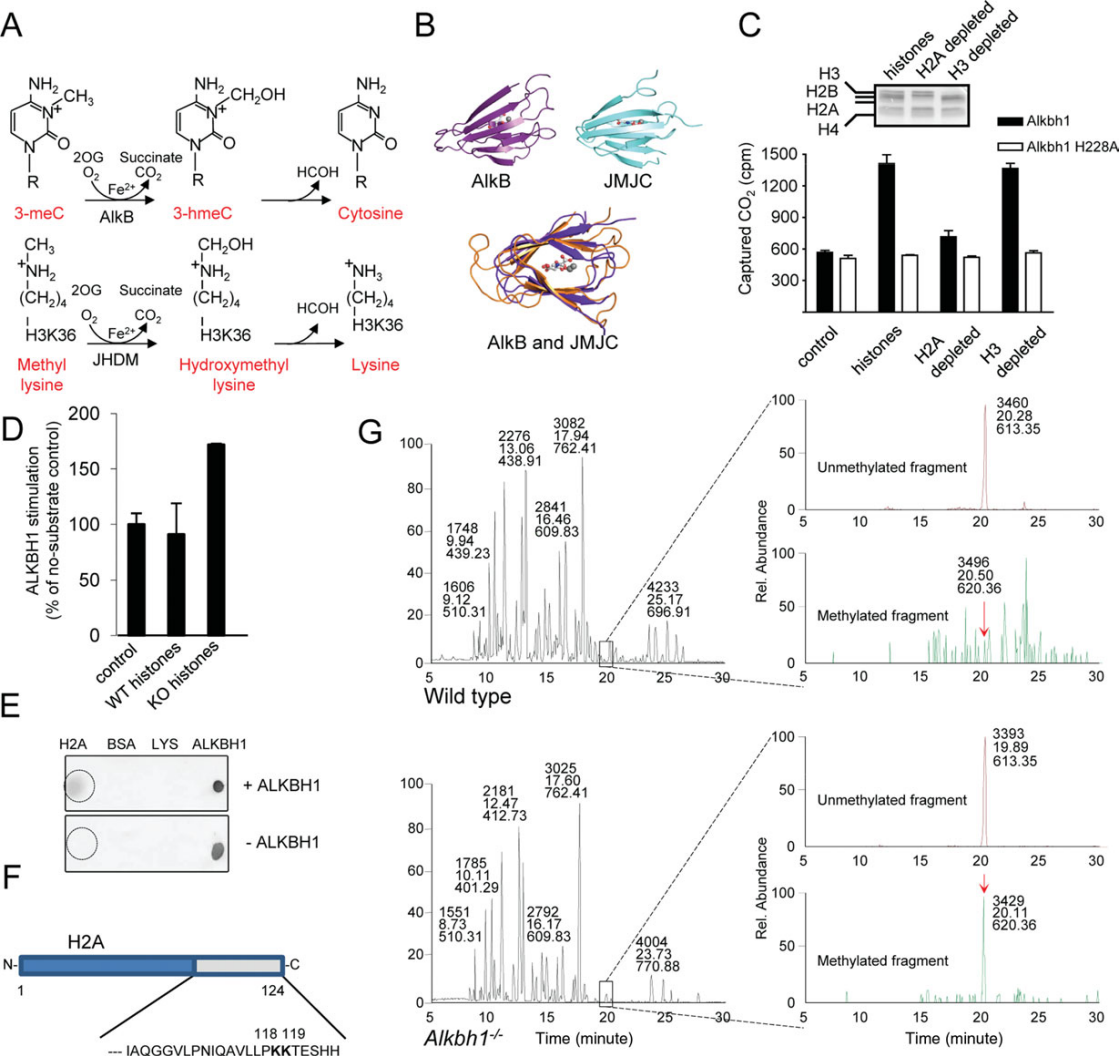
Histone H2A from *Alkbh1^−/−^* mouse embryonic fibroblast (MEF) cells contains a methylation group not present in wild-type (WT) histones. **(A):** Analogous hydroxylation mechanisms for demethylation of 3-methyladenine by the AlkB repair enzyme (top panel) and the hydroxylation of monomethyl lysine by a JmjC domain-containing histone demethylase, resulting in a similar loss of one methyl group and the generation of unmodified lysine (bottom panel). **(B):** The Fe(II)-2OG dioxygenase cores of *E. coli* AlkB (upper left) and the JmjC domain of human JMJD2A (upper right), shown in the same orientation. This shows the common “jellyroll” structural fold of the AlkB and JmjC-domain superfamilies. **(C):** Dioxygenase activity was evaluated by the CO_2_-capture assay using purified ALKBH1 with either purified HeLa core histones or core histones that were immunodepleted of H2A or H3. The ALKBH1 H228A variant is an inactive protein harboring a mutation in the Fe(II)-binding domain. Data are presented as the mean of two replicates. Error bars represent one SD. We used Coomassie-stained SDS-PAGE to analyze HeLa core histones immunodepleted for histones H2A and H3. **(D):** Histones purified from WT and *Alkbh1^−/−^* mESC show that *Alkbh1^−/−^* histones stimulate ALKBH1 activity approximately 80% more efficiently than WT histones. **(E):** Dot blot showing the physical interaction between the ALKBH1 and H2A proteins in vitro. **(F):** Representation of histone H2A, highlighting the C-terminal sequence harboring the region targeted by ALKBH1. **(G):** Analysis of histone H2A purified from WT or *Alkbh1^−/−^* MEFs using mass spectrometry. The maintenance of modifications after purification is shown in Supporting Information Figure S3A. Chromatograms on the left represent signals obtained from the entire run. Chromatograms on the right represent signals found for the peptide indicated in (E). The signal for the unmethylated peptides (upper chromatograms of the WT or *Alkbh1*^−/−^, respectively) is 6.68 × 10^5^ for WT MEFs and 4.30 × 10^5^ for *Alkbh1^−/−^* MEFs. The lower chromatograms represent the same peptide with the addition of 28.0314 Da (the mass of a dimethyl group or two monomethyl groups). The methylated peptide was undetectable in the WT sample, whereas there was a signal of 1.87 × 10^4^ for the *Alkbh1^−/−^* sample. The *x*-axis represents the elution time and the *y*-axis indicates the relative abundances of the peptides. Abbreviation: ALKBH1, AlkB homolog 1.

The GO analysis revealed that the majority of the human genes occupied by ALKBH1 are involved in early neural development (Fig. 5D). Aberrant expression of many of these genes results in neural tube defects [34]. Our data show that ALKBH1 binds to several genes essential for normal neurulation, including those that encode transcription factors (*YXB1* and *CREBB*), intermediates in signaling pathways such Wnt signaling (frizzled homolog 3 [*FZD3*]), and regulators of the cytoskeleton (*DYNC2H1*) and apoptosis (*KDM2B* and *APAF1*). To elucidate the role of ALKBH1 in regulation of these genes, we studied four genes in further detail, two associated with early development (rho-interacting, serine/threonine kinase 21 [*CIT*] and KDM2B lysine [K]-specific demethylase 2B [*KDM2B*]) [35, 36] and two at a later developmental stage (*FZD3* and roundabout homolog 1 [*ROBO1*]) [37, 38]. WT and *Alkbh1^−/−^* mESCs, differentiated in the presence of retinoic acid for 12 days, revealed a delayed induction of the genes involved in late development, *FZD3* and *ROBO1*. On the contrary, *CIT* and *KDM2B*, which are involved in early development, were expressed at a higher level in undifferentiated cells and maintained an increased expression upon differentiation (Fig. 5E). The former pattern of expression resembles the expression of differentiation markers (Fig. 2G) while the latter expression pattern is similar to genes involved in pluripotency (Fig. 2F). Together our results suggest that a subset of transcripts is repressed by ALKBH1, while another set is induced. The repressed genes are involved in pluripotency and early differentiation, while the induced genes are required later during development.

### Histone H2A from *Alkbh1*^−/−^ Cells Contains a Methylation Group Not Present in WT Histones

The *E. coli* AlkB and related mammalian dioxygenases oxidize their preferred substrates by decarboxylating 2OG to form succinate and CO_2_ (Fig. 6A). ALKBH1 is the mammalian AlkB homolog most similar in sequence to *E. coli* AlkB [39, 40]. Both *E. coli* AlkB and ALKBH1 are closely related to the JmjC domain-containing family proteins, which also contain a domain that binds both 2OG and Fe(II) [24]. The JmjC proteins are histone demethylases, and the JmjC domain of JmjD2 and *E. coli* AlkB is similar, particularly in the core domain that binds 2OG and Fe(II) (Fig. 6B). This similarity indicates that ALKBH1 may also function as a histone dioxygenase. While this article was in preparation, two studies suggested histone H2A dioxygenase activity of the *Schizosaccharomyces pombe* protein Ofd2 [41, 42]. Ofd2 is closely related to the human ALKBH1 protein.

To determine whether ALKBH1 has dioxygenase activity toward histones, we purified core histones from HeLa cells and incubated them with ALKBH1 in a CO_2_ capture assay (Fig. 6C). We identified a substantial increase in CO_2_ release when ALKBH1 was incubated with histones from HeLa cells relative to a control without substrate. The residues involved in binding to Fe(II) are well conserved in the AlkB homologs and are essential for catalytic activity [43]. To test whether the dioxygenase domain of ALKBH1 is required for activity, we mutated one of the iron-binding histidine residues to alanine and incubated the ALKBH1 H228A protein with HeLa histones. The release of CO_2_ was completely abolished, thereby confirming that the dioxygenase domain is required for the activity observed on histones from HeLa cells. To determine whether H2A was the preferred substrate, we immunodepleted H2A or H3 from HeLa histones and tested these samples using the CO_2_ capture assay (Fig. 6C). A substantial reduction in the levels of captured CO_2_ was detected when incubating ALKBH1 with H2A-depleted HeLa core histones, whereas histone H3 depletion did not affect CO_2_ capture. These results confirmed the dioxygenase screen that showed H2A is a substrate for ALKBH1. A final experiment using purified histones from WT or *Alkbh1^−/−^* mESCs revealed that *Alkbh1^−/−^* histones stimulated ALKBH1 activity approximately 80% more than histones purified from WT mESCs (Fig. 6D).

Along with the phenotype of *Alkbh1^−/−^* mice, our in vitro data support a role for ALKBH1 in epigenetic regulation and suggest that histone H2A is the substrate of ALKBH1 in vivo. To further substantiate this proposal, we used dot-blotting to demonstrate physical interaction between H2A and human ALKBH1 in vitro (Fig. 6E). As expected, ALKBH1 interacted with H2A and with the exception of a weak interaction with H4 did not interact with any of the other histones tested. The weak interaction with H4 may be explained by the high degree of homology between H2A and H4 (supporting information Fig. S4B). We next purified histone H2A, as well as histone H4 as a control, from WT and *Alkbh1^−/−^* mouse embryonic fibroblasts (MEFs) for subsequent mass spectrometry analyses. We verified that well-documented methyl groups on histones were not affected by the purification scheme used to prepare H2A and H4 (supporting information Fig. S4A). The patterns of post-translational modifications of H4 histones from WT and *Alkbh1^−/−^* MEFs were indistinguishable. By contrast, we identified different modifications on histone H2A preparations from WT and *Alkbh1^−/−^* MEFs (Fig. 6F, 6G). In *Alkbh1^−/−^* MEFs, a dimethyl group was present on histone H2A residues K118 or K119, whereas histone H2A purified from WT cells was devoid of methyl groups at these positions. In Figure 6G, the chromatograms on the left represent the signals obtained from the entire run. The chromatograms on the right represent signals from the indicated C-terminal peptide (IAQGGVLPNIQAVLLPKKTESHH). The signal intensities for the unmethylated peptides (Fig. 6F; upper chromatograms for the WT and *Alkbh1^−/−^*, respectively) were 6.68 × 10^5^ for WT and 4.30 × 10^5^ for *Alkbh1^−/−^*. The lower chromatograms represent the same peptide with the addition of 28.0314 Da (the mass of a dimethyl group or two monomethyl groups). In the WT sample, the methylated peptide was undetectable, whereas a signal of 1.87 × 10^4^ (approximately three to four times greater than background signal intensity) was present in the *Alkbh1^−/−^* sample. The same pattern was observed in multiple experiments. We conclude that *Alkbh1^−/−^* cells lack the enzyme to remove this methyl group. The amount of the methyl group is so low that mass spectrometry does not allow a firm confirmation of whether the dimethyl group is present on K118 or K119. We have not been able to confirm these results using methylated histone peptides, likely because the peptides lack the intrinsic structure of nucleosomes that this enzyme might require for function as seen for other histone demethylases [44]. Taken together, our in vitro and in vivo data suggest that ALKBH1 possesses dioxygenase activity specific for histone H2A.

## DISCUSSION

Here, we have shown that ALKBH1 binds to genes encoding developmental regulators, and we provide evidence that ALKBH1 is a histone dioxygenase that acts specifically on histone H2A. Moreover, we show that ALKBH1 is involved in the differentiation of mESCs along the neural lineage, and that lack of ALKBH1 increase the rate of apoptosis in differentiating mESCs. This correlates well with the lack of silencing of key pluripotency markers and a delayed induction of early differentiation genes in *Alkbh1^−/−^* mESCs.

### Alkbh1 Deficiency Delays Differentiation

The shutdown of the self-renewal machinery is a prerequisite for proper differentiation of ESCs. The protein NANOG is a central part of the regulatory transcriptional machinery in ESCs, where it sustains pluripotency. Forced expression of NANOG allows for autonomous self-renewal of cultured ESCs [4, 45]. *Alkbh1^−/−^* mESCs have elevated levels of *Nanog* transcripts relative to WT mESCs. Considering this, one would expect *Alkbh1^−/−^* mESCs to be refractory to differentiation. Upon exposure to retinoic acid, *Alkbh1^−/−^* mESCs differentiate and downregulate key markers of pluripotency, albeit later than in WT mESCs. At the same time, genes involved later in development revealed a delayed induction. The finely tuned balance between self-renewal and differentiation is governed by numerous interacting signaling pathways, and the exact mechanism(s) by which *Alkbh1* deficiency induces increased *Nanog* expression awaits further investigation. Previously, it was shown that NANOG and OCT4 co-occupy the promoter of human *ALKBH1* [7], which suggests that ALKBH1 is required for pluripotency of mESCs and/or during early differentiation. Signaling pathways often take advantage of regulatory feedback loops. However, our ChIP-seq study did not identify *NANOG* or *OCT4* as genes directly bound and regulated by ALKBH1. The effect of *Alkbh1*-deficiency on the pluripotency markers *Nanog* and *Oct4* in mESCs is thus likely to be a result of downstream targets of ALKBH1 rather than a direct effect of ALKBH1 on *Nanog* and *Oct4*. Intriguingly, of the genes bound and regulated by ALKBH1 is the JmjC domain-containing histone demethylase KDM2B which is a histone H3K36 dimethyl-specific demethylase regulating cell proliferation, cell cycle, and apoptosis [46]. KDM2B cooperates with OCT4 and is involved in cell fate determination [47]. Moreover, it was recently shown that KDM2B promotes the generation of induced pluripotent stem cells through demethylation of gene promoters, thereby enhancing the activation of early responsive genes in reprogramming [48]. This latter finding leads to the intriguing speculation that inhibition of ALKBH1 activity may contribute to increased reprogramming efficiency as well. This, however, requires further investigation.

### ALKBH1 Is a Histone H2A Dioxygenase

Bioinformatics analysis reveals a substantial degree of homology (23% identity and 59% similarity) between human ALKBH1 and *E. coli* AlkB [49, 50]. Yet the enzymatic function and preferred substrate of ALKBH1 have remained enigmatic and diverse enzymatic functions of the ALKBH1 protein have been proposed. Partial rescue of an *E. colialkB* mutant overexpressing ALKBH1 upon exposure to SN2-type alkylating agents (such as methyl methanesulfonate) suggested a role in repair [40], although this result has not been reproduced by others. Whereas Aas et al. failed to reveal ALKBH1 activity on methylated substrates [49], ALKBH1 has been reported to possess an apurinic/apyrimidinic (AP) lyase activity [20] that is independent of the cofactors 2OG and Fe(II) and it is unaffected by mutation of the putative metal-binding motif. The authors suggest that ALKBH1 might have dual functions that enable it to contribute to both regulation and repair. Our data show that histones stimulate the activity of recombinant human ALKBH1, and that this activity depends on histone H2A. To further substantiate this finding, we purified total histones from fibroblasts derived from WT and *Alkbh1^−/−^* mice. Mass spectrometric analysis of these histones showed the presence of a dimethyl group in the *Alkbh1^−/−^* cells that was lacking in the WT cells. We speculate that this specificity could possibly be supplied by a histone methyl transferase that methylates these novel sites in histone H2A. We, and others, have been unable to confirm these finding in vitro using synthetic histone peptides. This suggests that the observed ALKBH1 activity may require additional cofactors or interacting protein partners. It may also be that ALKBH1 activity requires correct three-dimensional folding of histone H2A.

## CONCLUSION

Global mapping of human ALKBH1 binding sites in the ESC genome indicates that ALKBH1 is required for the differentiation of tissues originating from the ectodermal lineage. Differentially expressed genes in *Alkbh1^−/−^* and WT mESCs provide further evidence for the role of ALKBH1 in the development of the nervous system. These observations correspond well with the severe developmental abnormalities observed in *Alkbh1^−/−^* mice. Although aspects of pluripotency and differentiation differ between human and mouse ESCs, our data suggest that the ALKBH1-mediated regulation of the histone H2A methylation status is involved during the early stages of development in both mice and humans. This distribution is suggestive of a gene-related function for ALKBH1, presumably at the level of chromatin regulation through the epigenetic status of histone H2A K118 and/or K119.

## Supplementary material

Figure S1: A) Sequences of the primers used in (B) (B) PCR on genomic DNA purified from wild-type and Alkbh1^−^ mESCs confirmed the removal of the Alkbh1 gene. Expected size of fragments are 421 base pairs for WT and 897 base pairs for Alkbh1^−^ (C) qRT-PCR using an Alkbh1 specific TaqManR probe (Applied Biosystems, assay ID: Mm01296827_m1) showed no presence of the transcript in Alkbh1^−^ mESCs.

Figure S2: Differentiation of Alkbh1^−^ mESCs leads to decreased viability. Upon induction of differentiation by retinoic acid, the culture of Alkbh1^−^ mESCs showed reduced viability as compared to the WT mESCs from differentiation day 2.

Figure S3: ChIP-qPCR validation showing enrichment of four selected ALKBH1 binding regions.

Figure S4: (A) Representative western blots verify the presence of histone methyl modifications after acid extraction. (+ represents 0.5 μg calf thymus histones). The histones were purified from MEF cells cultured under standard conditions in DMEM with 10 % fetal bovine serum. (B) Dot blot immunobinding assay suggest a possible interaction between human ALKBH1 and histone H2A. 0.5 μg of histones were spotted onto nitrocellulose membranes followed by incubation with human ALKBH1. After washing, the remaining ALKBH1 were visualized using an anti-ALKBH1 antibody (Sigma cat. no. A8103)

## References

[1] Loebel DA, Watson CM, De Young RA (2003). Lineage choice and differentiation in mouse embryos and embryonic stem cells. Dev Biol.

[2] Smith AG (2001). Embryo-derived stem cells: Of mice and men. Annu Rev Cell Dev Biol.

[3] Bernstein BE, Mikkelsen TS, Xie X (2006). A bivalent chromatin structure marks key developmental genes in embryonic stem cells. Cell.

[4] Chambers I, Colby D, Robertson M (2003). Functional expression cloning of Nanog, a pluripotency sustaining factor in embryonic stem cells. Cell.

[5] Masui S, Nakatake Y, Toyooka Y (2007). Pluripotency governed by Sox2 via regulation of Oct3/4 expression in mouse embryonic stem cells. Nat Cell Biol.

[6] Niwa H, Miyazaki J, Smith AG (2000). Quantitative expression of Oct-3/4 defines differentiation, dedifferentiation or self-renewal of ES cells. Nat Genet.

[7] Boyer LA, Lee TI, Cole MF (2005). Core transcriptional regulatory circuitry in human embryonic stem cells. Cell.

[8] Zhou Q, Chipperfield H, Melton DA (2007). A gene regulatory network in mouse embryonic stem cells. Proc Natl Acad Sci USA.

[9] Loh YH, Yang L, Yang JC (2011). Genomic approaches to deconstruct pluripotency. Annu Rev Genomics Hum Genet.

[10] Falnes PO, Johansen RF, Seeberg E (2002). AlkB-mediated oxidative demethylation reverses DNA damage in *Escherichia coli*. Nature.

[11] Trewick SC, Henshaw TF, Hausinger RP (2002). Oxidative demethylation by *Escherichia coli* AlkB directly reverts DNA base damage. Nature.

[12] Falnes PO, Rognes T (2003). DNA repair by bacterial AlkB proteins. Res Microbiol.

[13] Dango S, Mosammaparast N, Sowa ME (2011). DNA unwinding by ASCC3 helicase is coupled to ALKBH3-dependent DNA alkylation repair and cancer cell proliferation. Mol Cell.

[14] Ougland R, Zhang CM, Liiv A (2004). AlkB restores the biological function of mRNA and tRNA inactivated by chemical methylation. Mol Cell.

[15] Ringvoll J, Nordstrand LM, Vagbo CB (2006). Repair deficient mice reveal mABH2 as the primary oxidative demethylase for repairing 1meA and 3meC lesions in DNA. EMBO J.

[16] Songe-Moller L, van den Born E, Leihne V (2010). Mammalian ALKBH8 possesses tRNA methyltransferase activity required for the biogenesis of multiple wobble uridine modifications implicated in translational decoding. Mol Cell Biol.

[17] van den Born E, Vagbo CB, Songe-Moller L (2011). ALKBH8-mediated formation of a novel diastereomeric pair of wobble nucleosides in mammalian tRNA. Nat Commun.

[18] Jia G, Fu Y, Zhao X (2011). N6-methyladenosine in nuclear RNA is a major substrate of the obesity-associated FTO. Nat Chem Biol.

[19] Westbye MP, Feyzi E, Aas PA (2008). Human AlkB homolog 1 is a mitochondrial protein that demethylates 3-methylcytosine in DNA and RNA. J Biol Chem.

[20] Muller TA, Meek K, Hausinger RP (2010). Human AlkB homologue 1 (ABH1) exhibits DNA lyase activity at abasic sites. DNA Repair (Amst).

[21] Lee DH, Jin SG, Cai S (2005). Repair of methylation damage in DNA and RNA by mammalian AlkB homologues. J Biol Chem.

[22] Pan Z, Sikandar S, Witherspoon M (2008). Impaired placental trophoblast lineage differentiation in Alkbh1(−/−) mice. Dev Dyn.

[23] Tsujikawa K, Koike K, Kitae K (2007). Expression and sub-cellular localization of human ABH family molecules. J Cell Mol Med.

[24] Tsukada Y, Fang J, Erdjument-Bromage H (2006). Histone demethylation by a family of JmjC domain-containing proteins. Nature.

[25] Sedgwick B, Robins P, Lindahl T (2006). Direct removal of alkylation damage from DNA by AlkB and related DNA dioxygenases. Methods Enzymol.

[26] Nordstrand LM, Svard J, Larsen E (2010). Mice lacking Alkbh1 display sex-ratio distortion and unilateral eye defects. PLoS One.

[27] Schmitz SU, Albert M, Malatesta M (2011). Jarid1b targets genes regulating development and is involved in neural differentiation. EMBO J.

[28] Young RA (2011). Control of the embryonic stem cell state. Cell.

[29] Bergsland M, Werme M, Malewicz M (2006). The establishment of neuronal properties is controlled by Sox4 and Sox11. Genes Dev.

[30] Bhattaram P, Penzo-Mendez A, Sock E (2010). Organogenesis relies on SoxC transcription factors for the survival of neural and mesenchymal progenitors. Nat Commun.

[31] Thein DC, Thalhammer JM, Hartwig AC (2010). The closely related transcription factors Sox4 and Sox11 function as survival factors during spinal cord development. J Neurochem.

[32] Kenyon KL, Zaghloul N, Moody SA (2001). Transcription factors of the anterior neural plate alter cell movements of epidermal progenitors to specify a retinal fate. Dev Biol.

[33] Kuan CY, Roth KA, Flavell RA (2000). Mechanisms of programmed cell death in the developing brain. Trends Neurosci.

[34] Harris MJ, Juriloff DM (2007). Mouse mutants with neural tube closure defects and their role in understanding human neural tube defects. Birth Defects Res A Clin Mol Teratol.

[35] Cogswell CA, Sarkisian MR, Leung V (1998). A gene essential to brain growth and development maps to the distal arm of rat chromosome 12. Neurosci Lett.

[36] Fukuda T, Tokunaga A, Sakamoto R (2011). Fbxl10/Kdm2b deficiency accelerates neural progenitor cell death and leads to exencephaly. Mol Cell Neurosci.

[37] Armstrong A, Ryu YK, Chieco D (2011). Frizzled3 is required for neurogenesis and target innervation during sympathetic nervous system development. J Neurosci.

[38] Spitzweck B, Brankatschk M, Dickson BJ (2010). Distinct protein domains and expression patterns confer divergent axon guidance functions for Drosophila Robo receptors. Cell.

[39] Kataoka H, Yamamoto Y, Sekiguchi M (1983). A new gene (alkB) of Escherichia coli that controls sensitivity to methyl methane sulfonate. J Bacteriol.

[40] Wei YF, Carter KC, Wang RP (1996). Molecular cloning and functional analysis of a human cDNA encoding an *Escherichia coli* AlkB homolog, a protein involved in DNA alkylation damage repair. Nucleic Acids Res.

[41] Korvald H, Molstad Moe AM, Cederkvist FH (2011). *Schizosaccharomyces pombe* Ofd2 is a nuclear 2-oxoglutarate and iron dependent dioxygenase interacting with histones. PLoS One.

[42] Lando D, Balmer J, Laue ED (2012). The *S. pombe* histone H2A dioxygenase Ofd2 regulates gene expression during hypoxia. PLoS One.

[43] Sundheim O, Vagbo CB, Bjoras M (2006). Human ABH3 structure and key residues for oxidative demethylation to reverse DNA/RNA damage. EMBO J.

[44] Li Q, Zhou H, Wurtele H (2008). Acetylation of histone H3 lysine 56 regulates replication-coupled nucleosome assembly. Cell.

[45] Mitsui K, Tokuzawa Y, Itoh H (2003). The homeoprotein Nanog is required for maintenance of pluripotency in mouse epiblast and ES cells. Cell.

[46] He J, Kallin EM, Tsukada Y (2008). The H3K36 demethylase Jhdm1b/Kdm2b regulates cell proliferation and senescence through p15(Ink4b). Nat Struct Mol Biol.

[47] Wang T, Chen K, Zeng X (2011). The histone demethylases Jhdm1a/1b enhance somatic cell reprogramming in a vitamin-C-dependent manner. Cell Stem Cell.

[48] Liang G, He J, Zhang Y (2012). Kdm2b promotes induced pluripotent stem cell generation by facilitating gene activation early in reprogramming. Nat Cell Biol.

[49] Aas PA, Otterlei M, Falnes PO (2003). Human and bacterial oxidative demethylases repair alkylation damage in both RNA and DNA. Nature.

[50] Dinglay S, Trewick SC, Lindahl T (2000). Defective processing of methylated single-stranded DNA by *E. coli* AlkB mutants. Genes Dev.

[51] Bryja V, Bonilla S, Arenas E (2006). Derivation of mouse embryonic stem cells. Nat Protoc.

[52] Kivirikko KI, Myllyla R (1982). Posttranslational enzymes in the biosynthesis of collagen: Intracellular enzymes. Methods Enzymol.

[53] Dahl JA, Collas P (2008). A rapid micro chromatin immunoprecipitation assay (microChIP). Nat Protoc.

[54] Shechter D, Dormann HL, Allis CD (2007). Extraction, purification and analysis of histones. Nat Protoc.

