# Supplementary figures and images for: ALKBH1 Is a Histone H2A Dioxygenase Involved in Neural Differentiation
Source: Stem Cells. 2012 Sep 7;30(12):2672–82. doi: 10.1002/stem.1228 (PMC3546389; doi:10.1002/stem.1228)

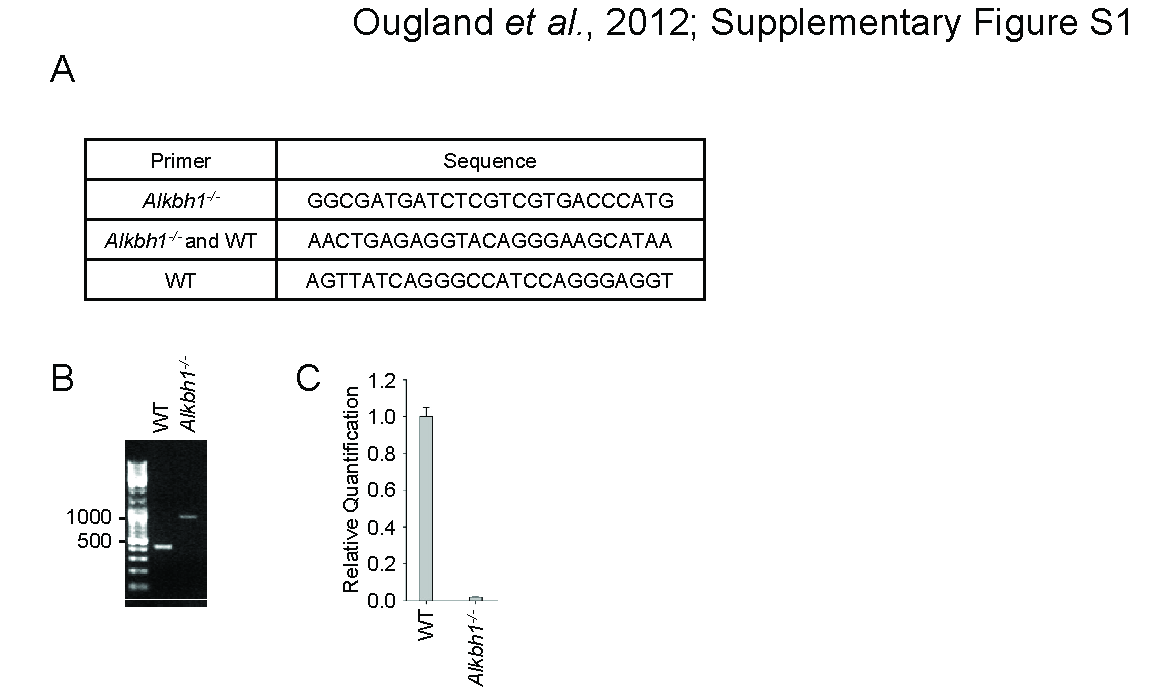

Supplement: Supplementary file 1 [file stem0030-2672-SD1.tif]

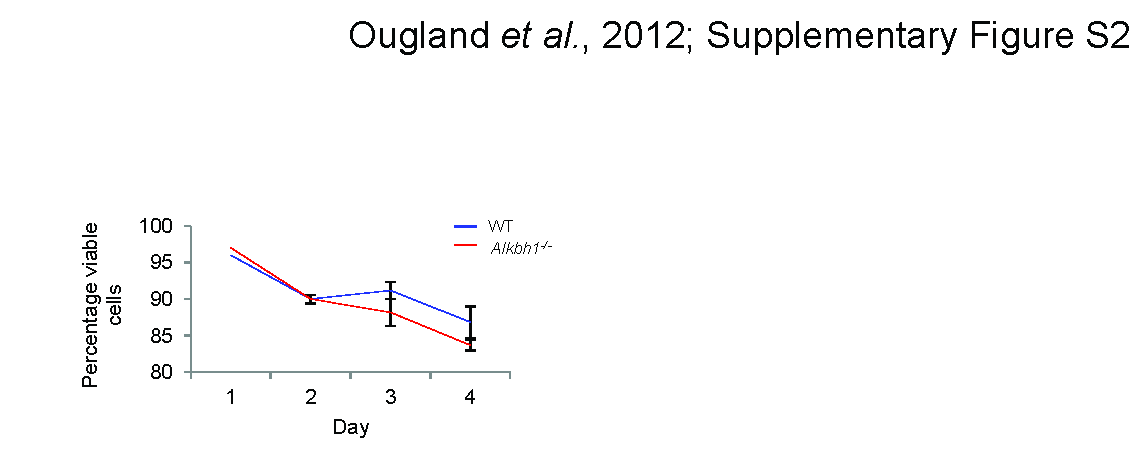

Supplement: Supplementary file 2 [file stem0030-2672-SD2.tif]

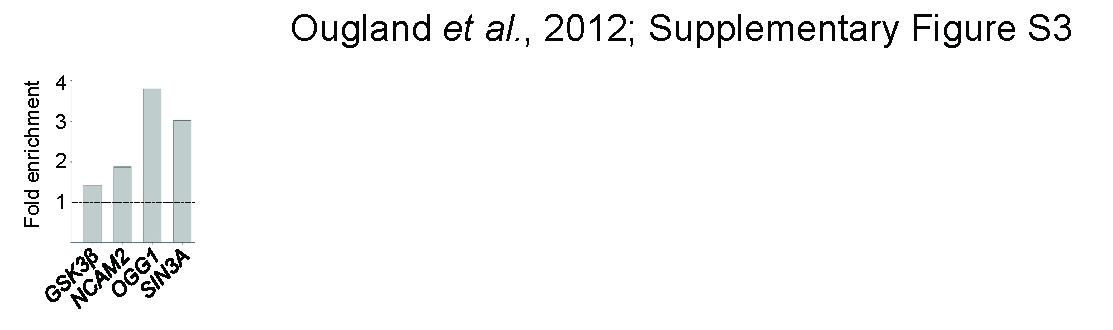

Supplement: Supplementary file 3 [file stem0030-2672-SD3.tif]

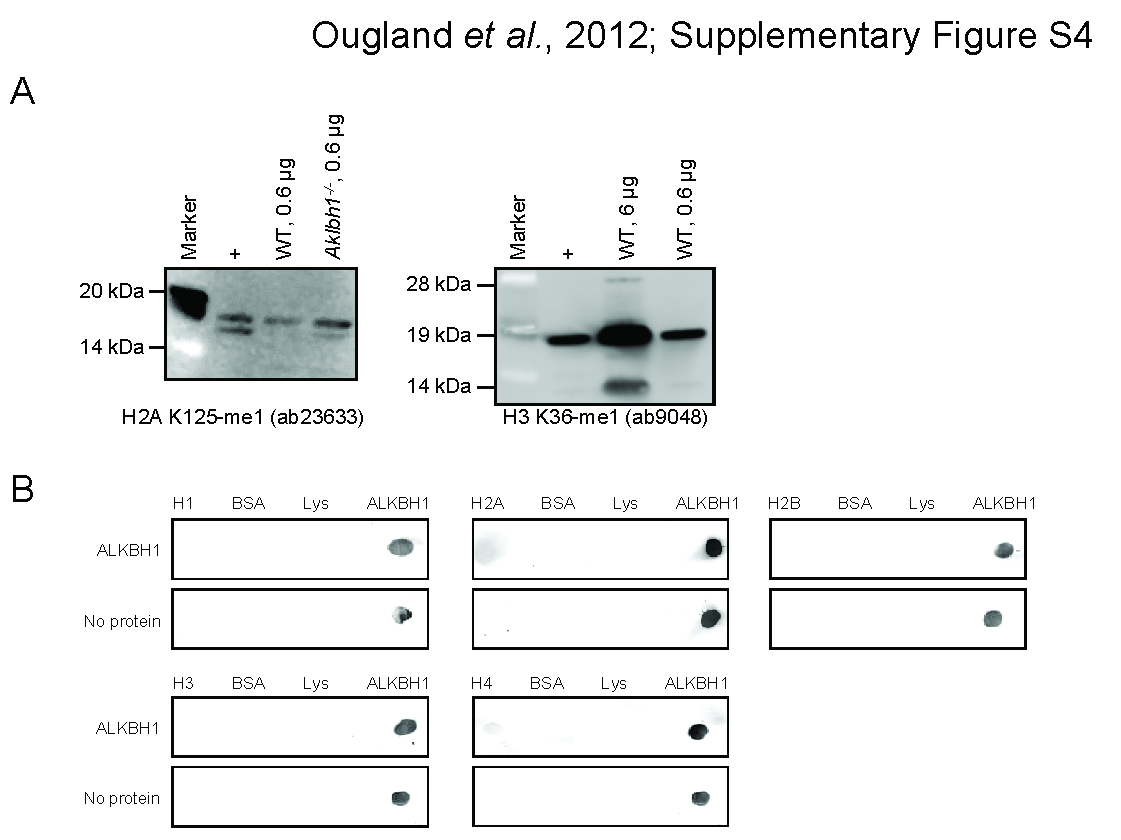

Supplement: Supplementary file 4 [file stem0030-2672-SD4.tif]
